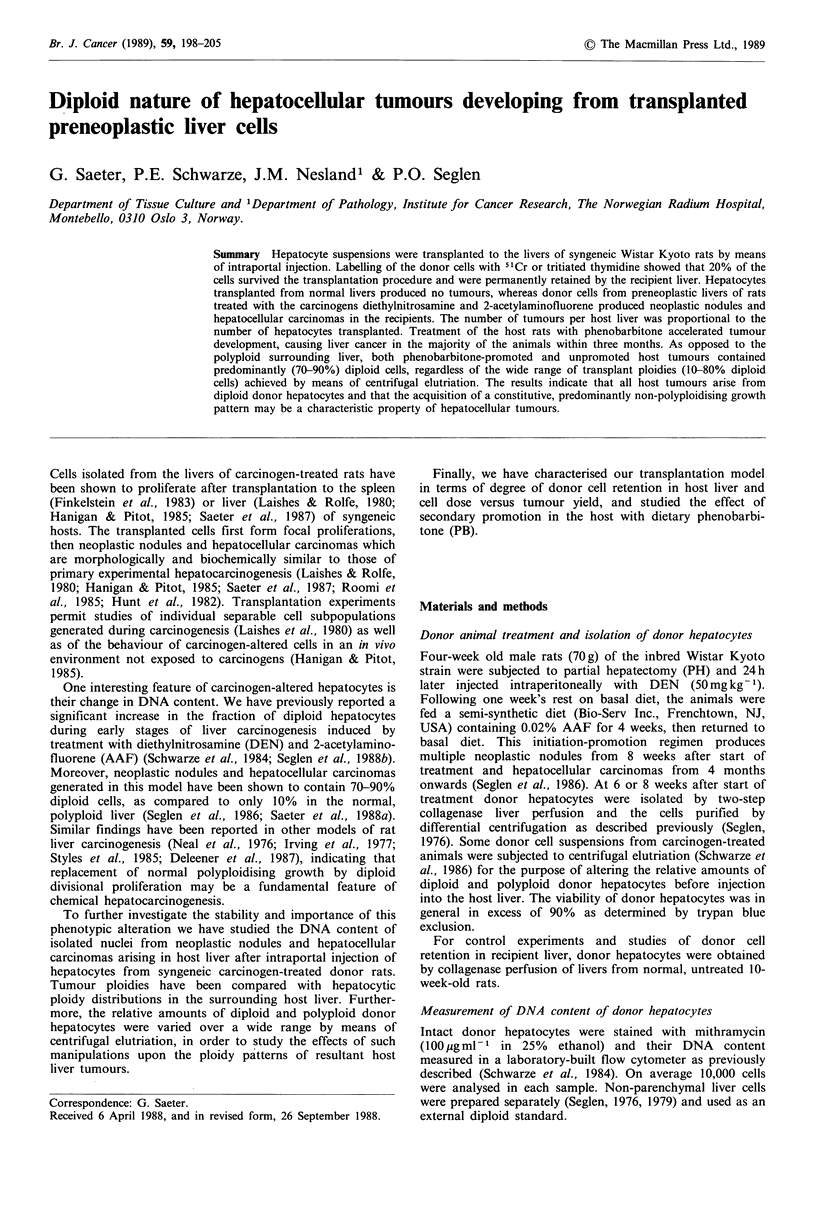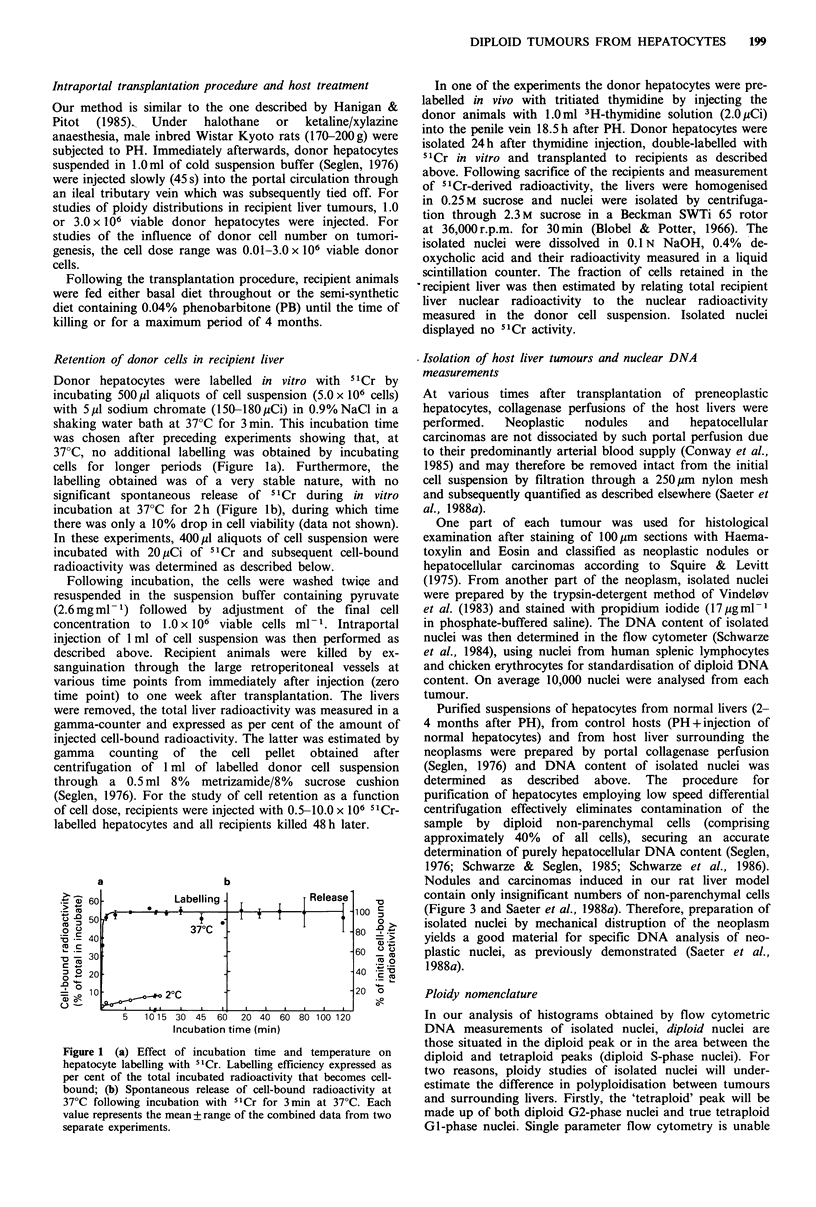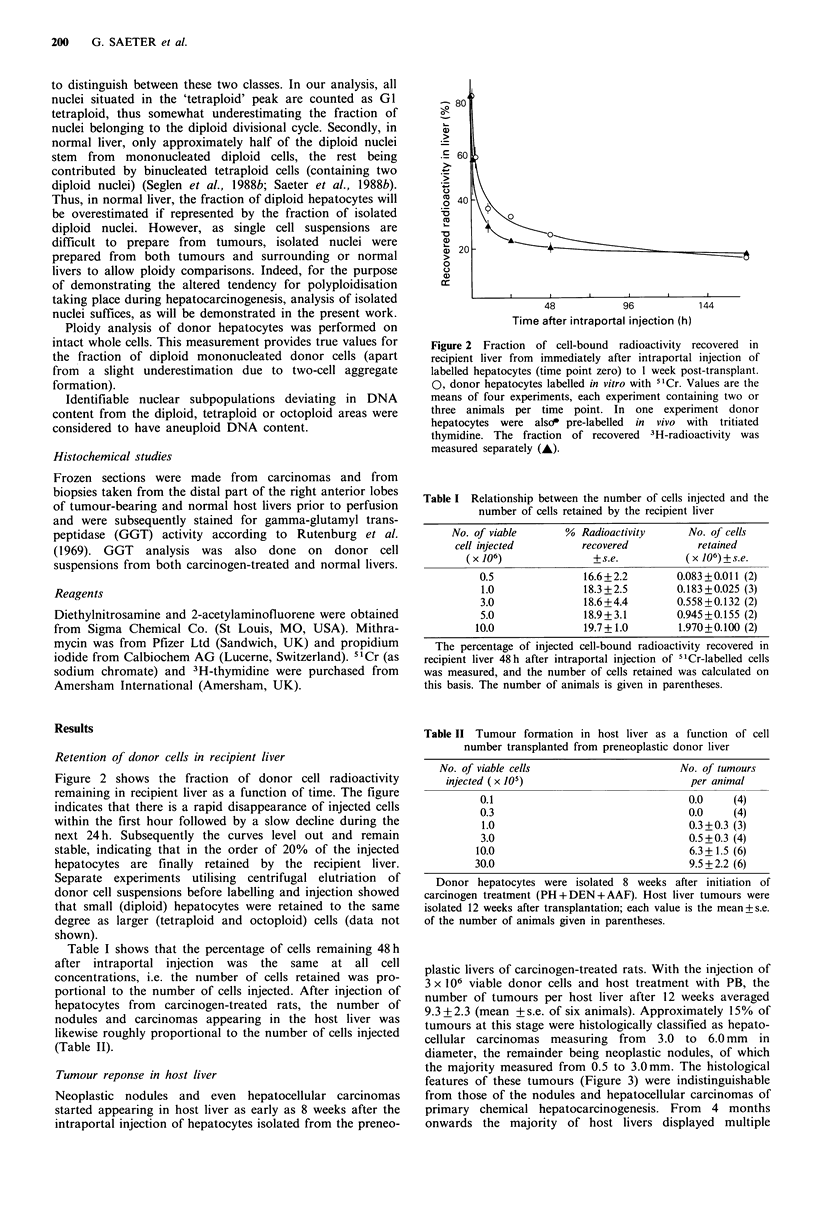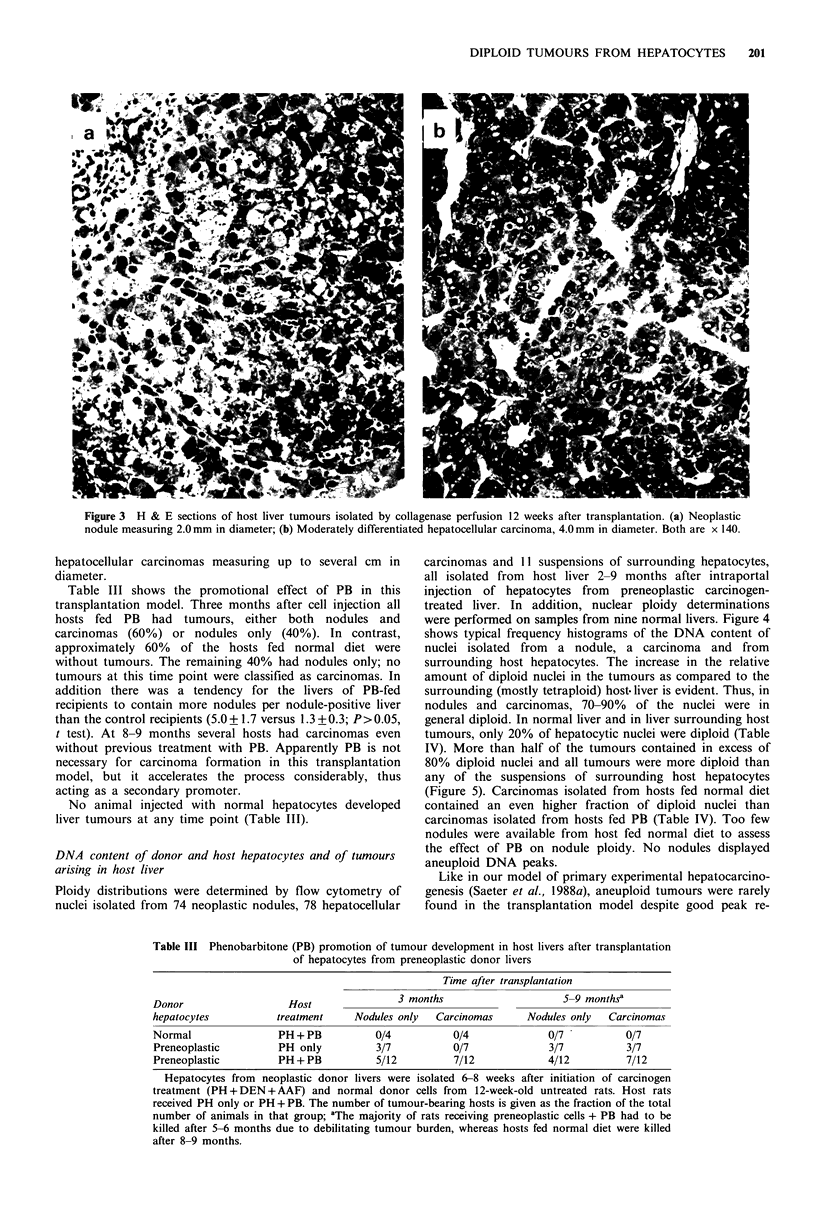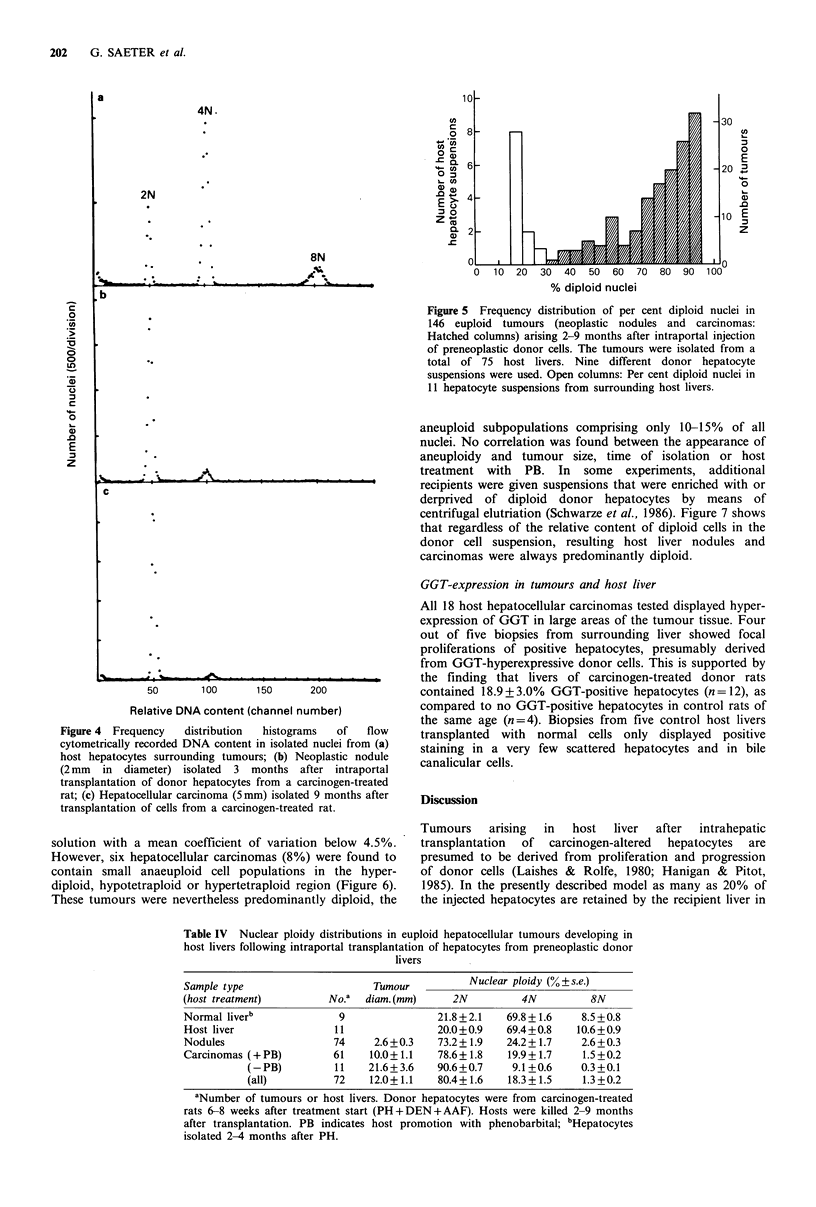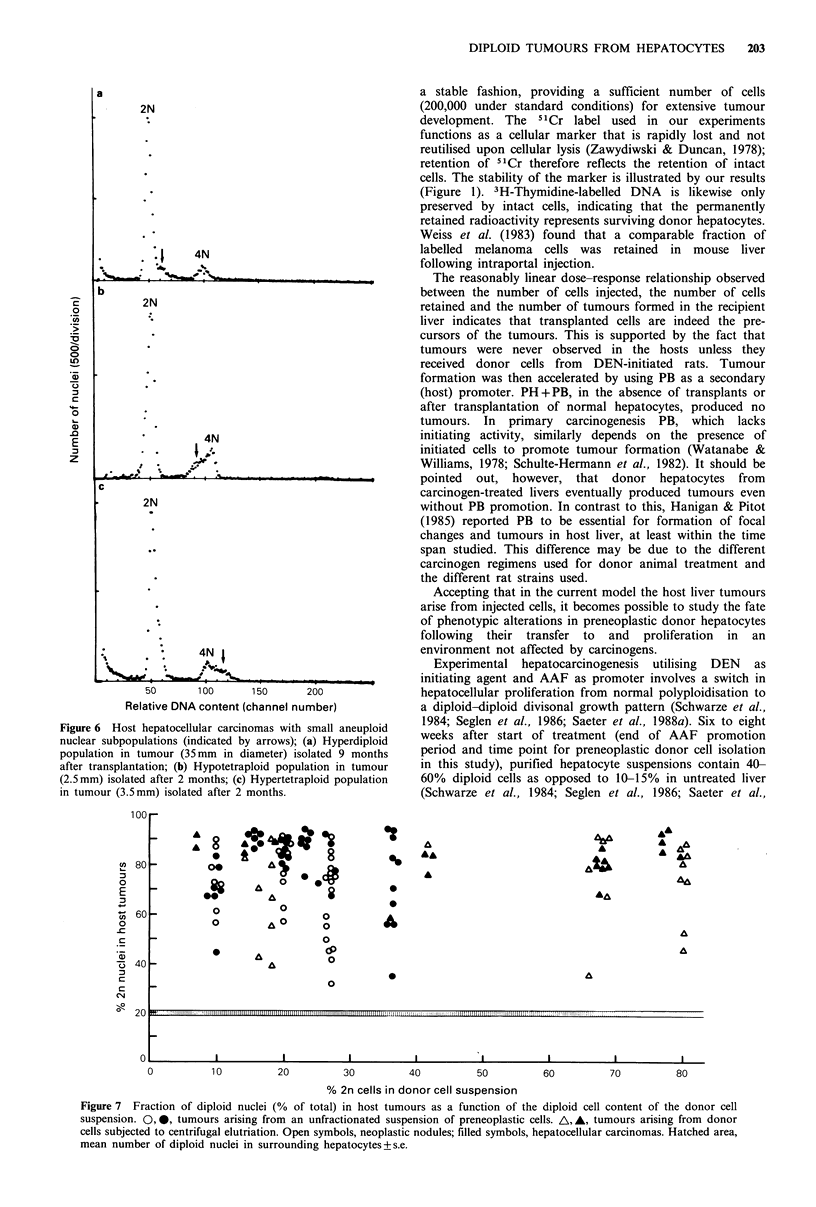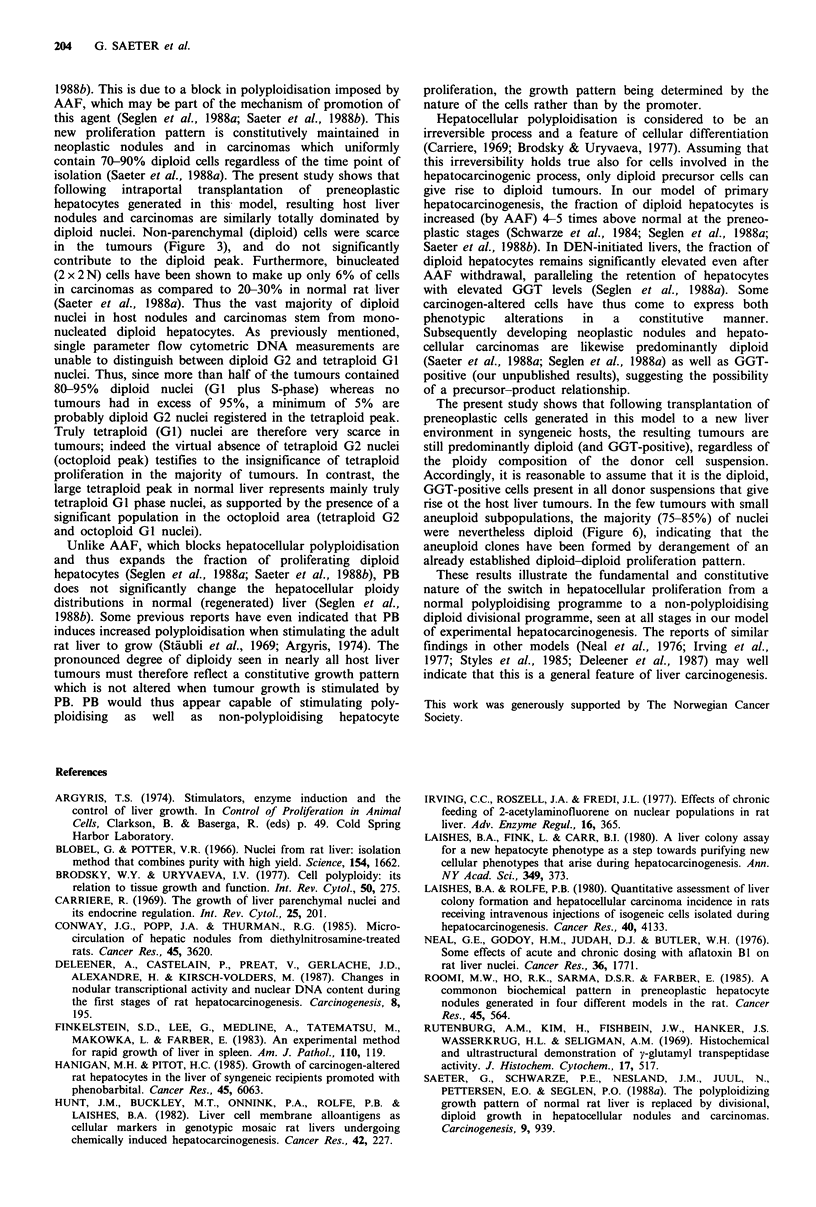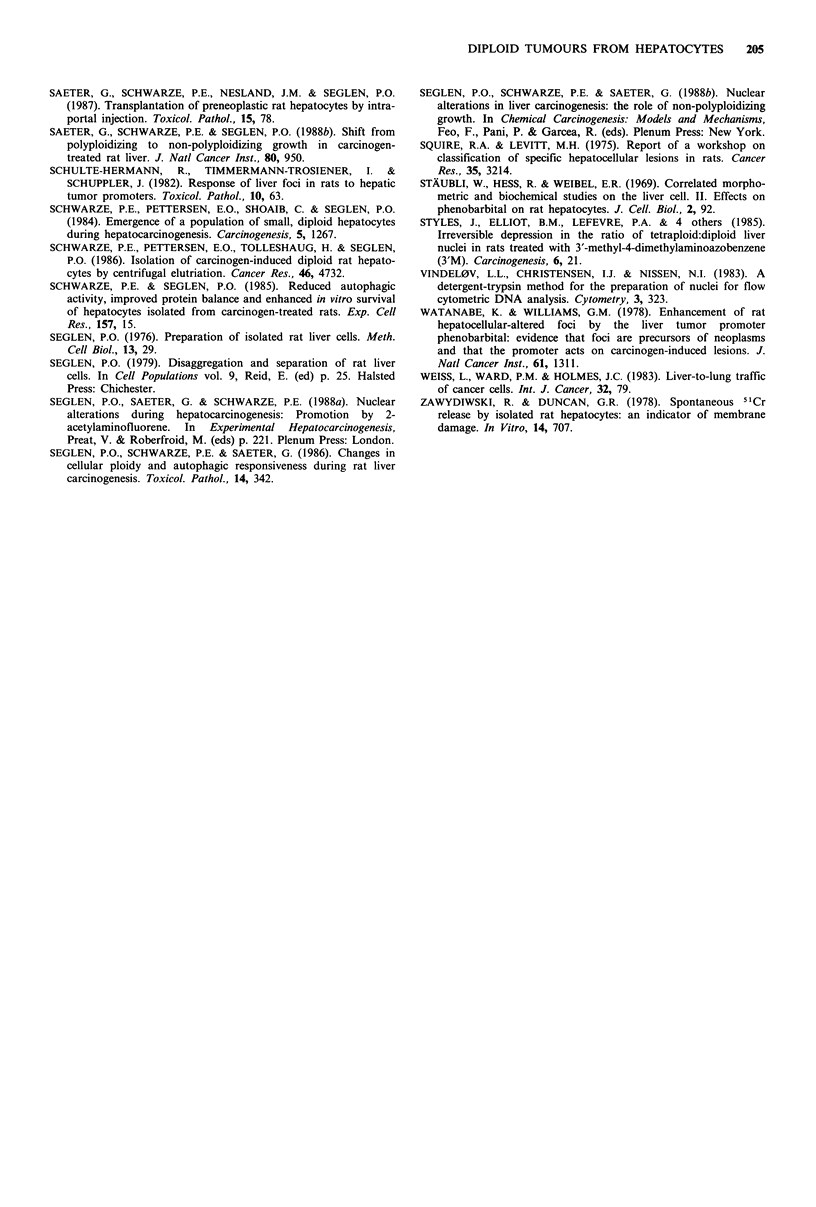# Diploid nature of hepatocellular tumours developing from transplanted preneoplastic liver cells.

**DOI:** 10.1038/bjc.1989.41

**Published:** 1989-02

**Authors:** G. Saeter, P. E. Schwarze, J. M. Nesland, P. O. Seglen

**Affiliations:** Department of Tissue Culture, Institute for Cancer Research, Norwegian Radium Hospital, Oslo.

## Abstract

**Images:**


*Br. J. Cancer* (1989), **59**, 198–205 © The Macmillan Press Ltd., 1989

### Diploid nature of hepatocellular tumours developing from transplanted preneoplastic liver cells

G. Saeter, P.E. Schwarze, J.M. Nesland[1] & P.O. Seglen

*Department of Tissue Culture and [1]Department of Pathology, Institute for Cancer Research, The Norwegian Radium Hospital, Montebello, 0310 Oslo 3, Norway.*

**Summary** Hepatocyte suspensions were transplanted to the livers of syngeneic Wistar Kyoto rats by means of intraportal injection. Labelling of the donor cells with $^{51}Cr$ or tritiated thymidine showed that 20% of the cells survived the transplantation procedure and were permanently retained by the recipient liver. Hepatocytes transplanted from normal livers produced no tumours, whereas donor cells from preneoplastic livers of rats treated with the carcinogens diethylnitrosamine and 2-acetylaminofluorene produced neoplastic nodules and hepatocellular carcinomas in the recipients. The number of tumours per host liver was proportional to the number of hepatocytes transplanted. Treatment of the host rats with phenobarbitone accelerated tumour development, causing liver cancer in the majority of the animals within three months. As opposed to the polyploid surrounding liver, both phenobarbitone-promoted and unpromoted host tumours contained predominantly (70–90%) diploid cells, regardless of the wide range of transplant ploidies (10–80% diploid cells) achieved by means of centrifugal elutriation. The results indicate that all host tumours arise from diploid donor hepatocytes and that the acquisition of a constitutive, predominantly non-polyploidising growth pattern may be a characteristic property of hepatocellular tumours.

Cells isolated from the livers of carcinogen-treated rats have been shown to proliferate after transplantation to the spleen (Finkelstein *et al.*, 1983) or liver (Laishes & Rolfe, 1980; Hanigan & Pitot, 1985; Saeter *et al.*, 1987) of syngeneic hosts. The transplanted cells first form focal proliferations, then neoplastic nodules and hepatocellular carcinomas which are morphologically and biochemically similar to those of primary experimental hepatocarcinogenesis (Laishes & Rolfe, 1980; Hanigan & Pitot, 1985; Saeter *et al.*, 1987; Roomi *et al.*, 1985; Hunt *et al.*, 1982). Transplantation experiments permit studies of individual separable cell subpopulations generated during carcinogenesis (Laishes *et al.*, 1980) as well as of the behaviour of carcinogen-altered cells in an *in vivo* environment not exposed to carcinogens (Hanigan & Pitot, 1985).

One interesting feature of carcinogen-altered hepatocytes is their change in DNA content. We have previously reported a significant increase in the fraction of diploid hepatocytes during early stages of liver carcinogenesis induced by treatment with diethylnitrosamine (DEN) and 2-acetylaminofluorene (AAF) (Schwarze *et al.*, 1984; Seglen *et al.*, 1988*b*). Moreover, neoplastic nodules and hepatocellular carcinomas generated in this model have been shown to contain 70–90% diploid cells, as compared to only 10% in the normal, polyploid liver (Seglen *et al.*, 1986; Saeter *et al.*, 1988*a*). Similar findings have been reported in other models of rat liver carcinogenesis (Neal *et al.*, 1976; Irving *et al.*, 1977; Styles *et al.*, 1985; Deleener *et al.*, 1987), indicating that replacement of normal polyploidising growth by diploid divisional proliferation may be a fundamental feature of chemical hepatocarcinogenesis.

To further investigate the stability and importance of this phenotypic alteration we have studied the DNA content of isolated nuclei from neoplastic nodules and hepatocellular carcinomas arising in host liver after intraportal injection of hepatocytes from syngeneic carcinogen-treated donor rats. Tumour ploidies have been compared with hepatocytic ploidy distributions in the surrounding host liver. Furthermore, the relative amounts of diploid and polyploid donor hepatocytes were varied over a wide range by means of centrifugal elutriation, in order to study the effects of such manipulations upon the ploidy patterns of resultant host liver tumours.

Correspondence: G. Saeter.
Received 6 April 1988, and in revised form, 26 September 1988.

Finally, we have characterised our transplantation model in terms of degree of donor cell retention in host liver and cell dose versus tumour yield, and studied the effect of secondary promotion in the host with dietary phenobarbitone (PB).

### Materials and methods

### *Donor animal treatment and isolation of donor hepatocytes*

Four-week old male rats (70 g) of the inbred Wistar Kyoto strain were subjected to partial hepatectomy (PH) and 24 h later injected intraperitoneally with DEN ($50\,mg\,kg^{-1}$). Following one week's rest on basal diet, the animals were fed a semi-synthetic diet (Bio-Serv Inc., Frenchtown, NJ, USA) containing 0.02% AAF for 4 weeks, then returned to basal diet. This initiation-promotion regimen produces multiple neoplastic nodules from 8 weeks after start of treatment and hepatocellular carcinomas from 4 months onwards (Seglen *et al.*, 1986). At 6 or 8 weeks after start of treatment donor hepatocytes were isolated by two-step collagenase liver perfusion and the cells purified by differential centrifugation as described previously (Seglen, 1976). Some donor cell suspensions from carcinogen-treated animals were subjected to centrifugal elutriation (Schwarze *et al.*, 1986) for the purpose of altering the relative amounts of diploid and polyploid donor hepatocytes before injection into the host liver. The viability of donor hepatocytes was in general in excess of 90% as determined by trypan blue exclusion.

For control experiments and studies of donor cell retention in recipient liver, donor hepatocytes were obtained by collagenase perfusion of livers from normal, untreated 10-week-old rats.

### *Measurement of DNA content of donor hepatocytes*

Intact donor hepatocytes were stained with mithramycin ($100\,\mu g\,ml^{-1}$ in 25% ethanol) and their DNA content measured in a laboratory-built flow cytometer as previously described (Schwarze *et al.*, 1984). On average 10,000 cells were analysed in each sample. Non-parenchymal liver cells were prepared separately (Seglen, 1976, 1979) and used as an external diploid standard.

*Intraportal transplantation procedure and host treatment*

Our method is similar to the one described by Hanigan & Pitot (1985). Under halothane or ketaline/xylazine anaesthesia, male inbred Wistar Kyoto rats (170–200 g) were subjected to PH. Immediately afterwards, donor hepatocytes suspended in 1.0 ml of cold suspension buffer (Seglen, 1976) were injected slowly (45 s) into the portal circulation through an ileal tributary vein which was subsequently tied off. For studies of ploidy distributions in recipient liver tumours, 1.0 or $3.0 \times 10^6$ viable donor hepatocytes were injected. For studies of the influence of donor cell number on tumorigenesis, the cell dose range was $0.01–3.0 \times 10^6$ viable donor cells.

Following the transplantation procedure, recipient animals were fed either basal diet throughout or the semi-synthetic diet containing 0.04% phenobarbitone (PB) until the time of killing or for a maximum period of 4 months.

*Retention of donor cells in recipient liver*

Donor hepatocytes were labelled *in vitro* with $^{51}Cr$ by incubating 500 $\mu$l aliquots of cell suspension ($5.0 \times 10^6$ cells) with 5 $\mu$l sodium chromate (150–180 $\mu$Ci) in 0.9% NaCl in a shaking water bath at 37°C for 3 min. This incubation time was chosen after preceding experiments showing that, at 37°C, no additional labelling was obtained by incubating cells for longer periods (Figure 1a). Furthermore, the labelling obtained was of a very stable nature, with no significant spontaneous release of $^{51}Cr$ during *in vitro* incubation at 37°C for 2 h (Figure 1b), during which time there was only a 10% drop in cell viability (data not shown). In these experiments, 400 $\mu$l aliquots of cell suspension were incubated with 20 $\mu$Ci of $^{51}Cr$ and subsequent cell-bound radioactivity was determined as described below.

Following incubation, the cells were washed twice and resuspended in the suspension buffer containing pyruvate (2.6 mg ml$^{-1}$) followed by adjustment of the final cell concentration to $1.0 \times 10^6$ viable cells ml$^{-1}$. Intraportal injection of 1 ml of cell suspension was then performed as described above. Recipient animals were killed by exsanguination through the large retroperitoneal vessels at various time points from immediately after injection (zero time point) to one week after transplantation. The livers were removed, the total liver radioactivity was measured in a gamma-counter and expressed as per cent of the amount of injected cell-bound radioactivity. The latter was estimated by gamma counting of the cell pellet obtained after centrifugation of 1 ml of labelled donor cell suspension through a 0.5 ml 8% metrizamide/8% sucrose cushion (Seglen, 1976). For the study of cell retention as a function of cell dose, recipients were injected with $0.5–10.0 \times 10^6$ $^{51}Cr$-labelled hepatocytes and all recipients killed 48 h later.

**Figure 1** (a) Effect of incubation time and temperature on hepatocyte labelling with $^{51}Cr$. Labelling efficiency expressed as per cent of the total incubated radioactivity that becomes cell-bound; (b) Spontaneous release of cell-bound radioactivity at 37°C following incubation with $^{51}Cr$ for 3 min at 37°C. Each value represents the mean ± range of the combined data from two separate experiments.

In one of the experiments the donor hepatocytes were prelabelled *in vivo* with tritiated thymidine by injecting the donor animals with 1.0 ml $^3H$-thymidine solution (2.0 $\mu$Ci) into the penile vein 18.5 h after PH. Donor hepatocytes were isolated 24 h after thymidine injection, double-labelled with $^{51}Cr$ *in vitro* and transplanted to recipients as described above. Following sacrifice of the recipients and measurement of $^{51}Cr$-derived radioactivity, the livers were homogenised in 0.25 M sucrose and nuclei were isolated by centrifugation through 2.3 M sucrose in a Beckman SWTi 65 rotor at 36,000 r.p.m. for 30 min (Blobel & Potter, 1966). The isolated nuclei were dissolved in 0.1 N NaOH, 0.4% deoxycholic acid and their radioactivity measured in a liquid scintillation counter. The fraction of cells retained in the recipient liver was then estimated by relating total recipient liver nuclear radioactivity to the nuclear radioactivity measured in the donor cell suspension. Isolated nuclei displayed no $^{51}Cr$ activity.

*Isolation of host liver tumours and nuclear DNA measurements*

At various times after transplantation of preneoplastic hepatocytes, collagenase perfusions of the host livers were performed. Neoplastic nodules and hepatocellular carcinomas are not dissociated by such portal perfusion due to their predominantly arterial blood supply (Conway *et al.*, 1985) and may therefore be removed intact from the initial cell suspension by filtration through a 250 $\mu$m nylon mesh and subsequently quantified as described elsewhere (Saeter *et al.*, 1988*a*).

One part of each tumour was used for histological examination after staining of 100 $\mu$m sections with Haematoxylin and Eosin and classified as neoplastic nodules or hepatocellular carcinomas according to Squire & Levitt (1975). From another part of the neoplasm, isolated nuclei were prepared by the trypsin-detergent method of Vindeløv *et al.* (1983) and stained with propidium iodide (17 $\mu$g ml$^{-1}$ in phosphate-buffered saline). The DNA content of isolated nuclei was then determined in the flow cytometer (Schwarze *et al.*, 1984), using nuclei from human splenic lymphocytes and chicken erythrocytes for standardisation of diploid DNA content. On average 10,000 nuclei were analysed from each tumour.

Purified suspensions of hepatocytes from normal livers (2–4 months after PH), from control hosts (PH + injection of normal hepatocytes) and from host liver surrounding the neoplasms were prepared by portal collagenase perfusion (Seglen, 1976) and DNA content of isolated nuclei was determined as described above. The procedure for purification of hepatocytes employing low speed differential centrifugation effectively eliminates contamination of the sample by diploid non-parenchymal cells (comprising approximately 40% of all cells), securing an accurate determination of purely hepatocellular DNA content (Seglen, 1976; Schwarze & Seglen, 1985; Schwarze *et al.*, 1986). Nodules and carcinomas induced in our rat liver model contain only insignificant numbers of non-parenchymal cells (Figure 3 and Saeter *et al.*, 1988*a*). Therefore, preparation of isolated nuclei by mechanical distruption of the neoplasm yields a good material for specific DNA analysis of neoplastic nuclei, as previously demonstrated (Saeter *et al.*, 1988*a*).

*Ploidy nomenclature*

In our analysis of histograms obtained by flow cytometric DNA measurements of isolated nuclei, *diploid* nuclei are those situated in the diploid peak or in the area between the diploid and tetraploid peaks (diploid S-phase nuclei). For two reasons, ploidy studies of isolated nuclei will underestimate the difference in polyploidisation between tumours and surrounding livers. Firstly, the 'tetraploid' peak will be made up of both diploid G2-phase nuclei and true tetraploid G1-phase nuclei. Single parameter flow cytometry is unable to distinguish between these two classes. In our analysis, all nuclei situated in the 'tetraploid' peak are counted as G1 tetraploid, thus somewhat underestimating the fraction of nuclei belonging to the diploid divisional cycle. Secondly, in normal liver, only approximately half of the diploid nuclei stem from mononucleated diploid cells, the rest being contributed by binucleated tetraploid cells (containing two diploid nuclei) (Seglen *et al.*, 1988*b*; Saeter *et al.*, 1988*b*). Thus, in normal liver, the fraction of diploid hepatocytes will be overestimated if represented by the fraction of isolated diploid nuclei. However, as single cell suspensions are difficult to prepare from tumours, isolated nuclei were prepared from both tumours and surrounding or normal livers to allow ploidy comparisons. Indeed, for the purpose of demonstrating the altered tendency for polyploidisation taking place during hepatocarcinogenesis, analysis of isolated nuclei suffices, as will be demonstrated in the present work.

Ploidy analysis of donor hepatocytes was performed on intact whole cells. This measurement provides true values for the fraction of diploid mononucleated donor cells (apart from a slight underestimation due to two-cell aggregate formation).

Identifiable nuclear subpopulations deviating in DNA content from the diploid, tetraploid or octoploid areas were considered to have aneuploid DNA content.

### *Histochemical studies*

Frozen sections were made from carcinomas and from biopsies taken from the distal part of the right anterior lobes of tumour-bearing and normal host livers prior to perfusion and were subsequently stained for gamma-glutamyl transpeptidase (GGT) activity according to Rutenburg *et al.* (1969). GGT analysis was also done on donor cell suspensions from both carcinogen-treated and normal livers.

### *Reagents*

Diethylnitrosamine and 2-acetylaminofluorene were obtained from Sigma Chemical Co. (St Louis, MO, USA). Mithramycin was from Pfizer Ltd (Sandwich, UK) and propidium iodide from Calbiochem AG (Lucerne, Switzerland). $^{51}Cr$ (as sodium chromate) and $^{3}H$-thymidine were purchased from Amersham International (Amersham, UK).

### Results

### *Retention of donor cells in recipient liver*

Figure 2 shows the fraction of donor cell radioactivity remaining in recipient liver as a function of time. The figure indicates that there is a rapid disappearance of injected cells within the first hour followed by a slow decline during the next 24 h. Subsequently the curves level out and remain stable, indicating that in the order of 20% of the injected hepatocytes are finally retained by the recipient liver. Separate experiments utilising centrifugal elutriation of donor cell suspensions before labelling and injection showed that small (diploid) hepatocytes were retained to the same degree as larger (tetraploid and octoploid) cells (data not shown).

Table I shows that the percentage of cells remaining 48 h after intraportal injection was the same at all cell concentrations, i.e. the number of cells retained was proportional to the number of cells injected. After injection of hepatocytes from carcinogen-treated rats, the number of nodules and carcinomas appearing in the host liver was likewise roughly proportional to the number of cells injected (Table II).

### *Tumour reponse in host liver*

Neoplastic nodules and even hepatocellular carcinomas started appearing in host liver as early as 8 weeks after the intraportal injection of hepatocytes isolated from the preneoplastic livers of carcinogen-treated rats. With the injection of $3 \times 10^6$ viable donor cells and host treatment with PB, the number of tumours per host liver after 12 weeks averaged $9.3 \pm 2.3$ (mean $\pm$s.e. of six animals). Approximately 15% of tumours at this stage were histologically classified as hepatocellular carcinomas measuring from 3.0 to 6.0 mm in diameter, the remainder being neoplastic nodules, of which the majority measured from 0.5 to 3.0 mm. The histological features of these tumours (Figure 3) were indistinguishable from those of the nodules and hepatocellular carcinomas of primary chemical hepatocarcinogenesis. From 4 months onwards the majority of host livers displayed multiple

**Figure 2** Fraction of cell-bound radioactivity recovered in recipient liver from immediately after intraportal injection of labelled hepatocytes (time point zero) to 1 week post-transplant. ○, donor hepatocytes labelled *in vitro* with $^{51}Cr$. Values are the means of four experiments, each experiment containing two or three animals per time point. In one experiment donor hepatocytes were also pre-labelled *in vivo* with tritiated thymidine. The fraction of recovered $^{3}H$-radioactivity was measured separately (▲).

**Table I** Relationship between the number of cells injected and the number of cells retained by the recipient liver

| *No. of viable cell injected* ($\times 10^6$) | *% Radioactivity recovered* $\pm$*s.e.* | *No. of cells retained* ($\times 10^6$)$\pm$*s.e.* |
|---|---|---|
| 0.5 | 16.6±2.2 | 0.083±0.011 (2) |
| 1.0 | 18.3±2.5 | 0.183±0.025 (3) |
| 3.0 | 18.6±4.4 | 0.558±0.132 (2) |
| 5.0 | 18.9±3.1 | 0.945±0.155 (2) |
| 10.0 | 19.7±1.0 | 1.970±0.100 (2) |

The percentage of injected cell-bound radioactivity recovered in recipient liver 48 h after intraportal injection of $^{51}Cr$-labelled cells was measured, and the number of cells retained was calculated on this basis. The number of animals is given in parentheses.

**Table II** Tumour formation in host liver as a function of cell number transplanted from preneoplastic donor liver

| *No. of viable cells injected* ($\times 10^5$) | *No. of tumours per animal* |
|---|---|
| 0.1 | 0.0 (4) |
| 0.3 | 0.0 (4) |
| 1.0 | 0.3±0.3 (3) |
| 3.0 | 0.5±0.3 (4) |
| 10.0 | 6.3±1.5 (6) |
| 30.0 | 9.5±2.2 (6) |

Donor hepatocytes were isolated 8 weeks after initiation of carcinogen treatment (PH+DEN+AAF). Host liver tumours were isolated 12 weeks after transplantation; each value is the mean±s.e. of the number of animals given in parentheses.

**Figure 3** H & E sections of host liver tumours isolated by collagenase perfusion 12 weeks after transplantation. (**a**) Neoplastic nodule measuring 2.0 mm in diameter; (**b**) Moderately differentiated hepatocellular carcinoma, 4.0 mm in diameter. Both are $\times 140$.

hepatocellular carcinomas measuring up to several cm in diameter.

Table III shows the promotional effect of PB in this transplantation model. Three months after cell injection all hosts fed PB had tumours, either both nodules and carcinomas (60%) or nodules only (40%). In contrast, approximately 60% of the hosts fed normal diet were without tumours. The remaining 40% had nodules only; no tumours at this time point were classified as carcinomas. In addition there was a tendency for the livers of PB-fed recipients to contain more nodules per nodule-positive liver than the control recipients ($5.0 \pm 1.7$ versus $1.3 \pm 0.3$; $P > 0.05$, $t$ test). At 8–9 months several hosts had carcinomas even without previous treatment with PB. Apparently PB is not necessary for carcinoma formation in this transplantation model, but it accelerates the process considerably, thus acting as a secondary promoter.

No animal injected with normal hepatocytes developed liver tumours at any time point (Table III).

### *DNA content of donor and host hepatocytes and of tumours arising in host liver*

Ploidy distributions were determined by flow cytometry of nuclei isolated from 74 neoplastic nodules, 78 hepatocellular carcinomas and 11 suspensions of surrounding hepatocytes, all isolated from host liver 2–9 months after intraportal injection of hepatocytes from preneoplastic carcinogen-treated liver. In addition, nuclear ploidy determinations were performed on samples from nine normal livers. Figure 4 shows typical frequency histograms of the DNA content of nuclei isolated from a nodule, a carcinoma and from surrounding host hepatocytes. The increase in the relative amount of diploid nuclei in the tumours as compared to the surrounding (mostly tetraploid) host liver is evident. Thus, in nodules and carcinomas, 70–90% of the nuclei were in general diploid. In normal liver and in liver surrounding host tumours, only 20% of hepatocytic nuclei were diploid (Table IV). More than half of the tumours contained in excess of 80% diploid nuclei and all tumours were more diploid than any of the suspensions of surrounding host hepatocytes (Figure 5). Carcinomas isolated from hosts fed normal diet contained an even higher fraction of diploid nuclei than carcinomas isolated from hosts fed PB (Table IV). Too few nodules were available from host fed normal diet to assess the effect of PB on nodule ploidy. No nodules displayed aneuploid DNA peaks.

Like in our model of primary experimental hepatocarcinogenesis (Saeter *et al.*, 1988*a*), aneuploid tumours were rarely found in the transplantation model despite good peak re-

**Table III** Phenobarbitone (PB) promotion of tumour development in host livers after transplantation of hepatocytes from preneoplastic donor livers

| *Donor hepatocytes* | *Host treatment* | *Time after transplantation* — *3 months*: *Nodules only* | *3 months*: *Carcinomas* | *5–9 months*[a]: *Nodules only* | *5–9 months*[a]: *Carcinomas* |
|---|---|---|---|---|---|
| Normal | PH + PB | 0/4 | 0/4 | 0/7 | 0/7 |
| Preneoplastic | PH only | 3/7 | 0/7 | 3/7 | 3/7 |
| Preneoplastic | PH + PB | 5/12 | 7/12 | 4/12 | 7/12 |

Hepatocytes from neoplastic donor livers were isolated 6–8 weeks after initiation of carcinogen treatment (PH + DEN + AAF) and normal donor cells from 12-week-old untreated rats. Host rats received PH only or PH + PB. The number of tumour-bearing hosts is given as the fraction of the total number of animals in that group; [a]The majority of rats receiving preneoplastic cells + PB had to be killed after 5–6 months due to debilitating tumour burden, whereas hosts fed normal diet were killed after 8–9 months.

**Figure 4** Frequency distribution histograms of flow cytometrically recorded DNA content in isolated nuclei from (**a**) host hepatocytes surrounding tumours; (**b**) Neoplastic nodule (2 mm in diameter) isolated 3 months after intraportal transplantation of donor hepatocytes from a carcinogen-treated rat; (**c**) Hepatocellular carcinoma (5 mm) isolated 9 months after transplantation of cells from a carcinogen-treated rat.

**Figure 5** Frequency distribution of per cent diploid nuclei in 146 euploid tumours (neoplastic nodules and carcinomas: Hatched columns) arising 2–9 months after intraportal injection of preneoplastic donor cells. The tumours were isolated from a total of 75 host livers. Nine different donor hepatocyte suspensions were used. Open columns: Per cent diploid nuclei in 11 hepatocyte suspensions from surrounding host livers.

solution with a mean coefficient of variation below 4.5%. However, six hepatocellular carcinomas (8%) were found to contain small anaeuploid cell populations in the hyperdiploid, hypotetraploid or hypertetraploid region (Figure 6). These tumours were nevertheless predominantly diploid, the aneuploid subpopulations comprising only 10–15% of all nuclei. No correlation was found between the appearance of aneuploidy and tumour size, time of isolation or host treatment with PB. In some experiments, additional recipients were given suspensions that were enriched with or derprived of diploid donor hepatocytes by means of centrifugal elutriation (Schwarze *et al.*, 1986). Figure 7 shows that regardless of the relative content of diploid cells in the donor cell suspension, resulting host liver nodules and carcinomas were always predominantly diploid.

### *GGT-expression in tumours and host liver*

All 18 host hepatocellular carcinomas tested displayed hyperexpression of GGT in large areas of the tumour tissue. Four out of five biopsies from surrounding liver showed focal proliferations of positive hepatocytes, presumably derived from GGT-hyperexpressive donor cells. This is supported by the finding that livers of carcinogen-treated donor rats contained 18.9±3.0% GGT-positive hepatocytes ($n=12$), as compared to no GGT-positive hepatocytes in control rats of the same age ($n=4$). Biopsies from five control host livers transplanted with normal cells only displayed positive staining in a very few scattered hepatocytes and in bile canalicular cells.

### Discussion

Tumours arising in host liver after intrahepatic transplantation of carcinogen-altered hepatocytes are presumed to be derived from proliferation and progression of donor cells (Laishes & Rolfe, 1980; Hanigan & Pitot, 1985). In the presently described model as many as 20% of the injected hepatocytes are retained by the recipient liver in

**Table IV** Nuclear ploidy distributions in euploid hepatocellular tumours developing in host livers following intraportal transplantation of hepatocytes from preneoplastic donor livers

| *Sample type* | | *Tumour* | *Nuclear ploidy (%±s.e.)* | | |
|---|---|---|---|---|---|
| *(host treatment)* | *No.*[a] | *diam. (mm)* | *2N* | *4N* | *8N* |
| Normal liver[b] | 9 | | 21.8±2.1 | 69.8±1.6 | 8.5±0.8 |
| Host liver | 11 | | 20.0±0.9 | 69.4±0.8 | 10.6±0.9 |
| Nodules | 74 | 2.6±0.3 | 73.2±1.9 | 24.2±1.7 | 2.6±0.3 |
| Carcinomas (+PB) | 61 | 10.0±1.1 | 78.6±1.8 | 19.9±1.7 | 1.5±0.2 |
| (−PB) | 11 | 21.6±3.6 | 90.6±0.7 | 9.1±0.6 | 0.3±0.1 |
| (all) | 72 | 12.0±1.1 | 80.4±1.6 | 18.3±1.5 | 1.3±0.2 |

[a]Number of tumours or host livers. Donor hepatocytes were from carcinogen-treated rats 6–8 weeks after treatment start (PH+DEN+AAF). Hosts were killed 2–9 months after transplantation. PB indicates host promotion with phenobarbital; [b]Hepatocytes isolated 2–4 months after PH.

**Figure 6** Host hepatocellular carcinomas with small aneuploid nuclear subpopulations (indicated by arrows); (**a**) Hyperdiploid population in tumour (35 mm in diameter) isolated 9 months after transplantation; (**b**) Hypotetraploid population in tumour (2.5 mm) isolated after 2 months; (**c**) Hypertetraploid population in tumour (3.5 mm) isolated after 2 months.

a stable fashion, providing a sufficient number of cells (200,000 under standard conditions) for extensive tumour development. The $^{51}Cr$ label used in our experiments functions as a cellular marker that is rapidly lost and not reutilised upon cellular lysis (Zawydiwski & Duncan, 1978); retention of $^{51}Cr$ therefore reflects the retention of intact cells. The stability of the marker is illustrated by our results (Figure 1). $^{3}H$-Thymidine-labelled DNA is likewise only preserved by intact cells, indicating that the permanently retained radioactivity represents surviving donor hepatocytes. Weiss *et al.* (1983) found that a comparable fraction of labelled melanoma cells was retained in mouse liver following intraportal injection.

The reasonably linear dose–response relationship observed between the number of cells injected, the number of cells retained and the number of tumours formed in the recipient liver indicates that transplanted cells are indeed the precursors of the tumours. This is supported by the fact that tumours were never observed in the hosts unless they received donor cells from DEN-initiated rats. Tumour formation was then accelerated by using PB as a secondary (host) promoter. PH + PB, in the absence of transplants or after transplantation of normal hepatocytes, produced no tumours. In primary carcinogenesis PB, which lacks initiating activity, similarly depends on the presence of initiated cells to promote tumour formation (Watanabe & Williams, 1978; Schulte-Hermann *et al.*, 1982). It should be pointed out, however, that donor hepatocytes from carcinogen-treated livers eventually produced tumours even without PB promotion. In contrast to this, Hanigan & Pitot (1985) reported PB to be essential for formation of focal changes and tumours in host liver, at least within the time span studied. This difference may be due to the different carcinogen regimens used for donor animal treatment and the different rat strains used.

Accepting that in the current model the host liver tumours arise from injected cells, it becomes possible to study the fate of phenotypic alterations in preneoplastic donor hepatocytes following their transfer to and proliferation in an environment not affected by carcinogens.

Experimental hepatocarcinogenesis utilising DEN as initiating agent and AAF as promoter involves a switch in hepatocellular proliferation from normal polyploidisation to a diploid–diploid divisonal growth pattern (Schwarze *et al.*, 1984; Seglen *et al.*, 1986; Saeter *et al.*, 1988*a*). Six to eight weeks after start of treatment (end of AAF promotion period and time point for preneoplastic donor cell isolation in this study), purified hepatocyte suspensions contain 40–60% diploid cells as opposed to 10–15% in untreated liver (Schwarze *et al.*, 1984; Seglen *et al.*, 1986; Saeter *et al.*,

**Figure 7** Fraction of diploid nuclei (% of total) in host tumours as a function of the diploid cell content of the donor cell suspension. ○, ●, tumours arising from an unfractionated suspension of preneoplastic cells. △, ▲, tumours arising from donor cells subjected to centrifugal elutriation. Open symbols, neoplastic nodules; filled symbols, hepatocellular carcinomas. Hatched area, mean number of diploid nuclei in surrounding hepatocytes ± s.e.

1988b). This is due to a block in polyploidisation imposed by AAF, which may be part of the mechanism of promotion of this agent (Seglen et al., 1988a; Saeter et al., 1988b). This new proliferation pattern is constitutively maintained in neoplastic nodules and in carcinomas which uniformly contain 70–90% diploid cells regardless of the time point of isolation (Saeter et al., 1988a). The present study shows that following intraportal transplantation of preneoplastic hepatocytes generated in this model, resulting host liver nodules and carcinomas are similarly totally dominated by diploid nuclei. Non-parenchymal (diploid) cells were scarce in the tumours (Figure 3), and do not significantly contribute to the diploid peak. Furthermore, binucleated (2 × 2 N) cells have been shown to make up only 6% of cells in carcinomas as compared to 20–30% in normal rat liver (Saeter et al., 1988a). Thus the vast majority of diploid nuclei in host nodules and carcinomas stem from mononucleated diploid hepatocytes. As previously mentioned, single parameter flow cytometric DNA measurements are unable to distinguish between diploid G2 and tetraploid G1 nuclei. Thus, since more than half of the tumours contained 80–95% diploid nuclei (G1 plus S-phase) whereas no tumours had in excess of 95%, a minimum of 5% are probably diploid G2 nuclei registered in the tetraploid peak. Truly tetraploid (G1) nuclei are therefore very scarce in tumours; indeed the virtual absence of tetraploid G2 nuclei (octoploid peak) testifies to the insignificance of tetraploid proliferation in the majority of tumours. In contrast, the large tetraploid peak in normal liver represents mainly truly tetraploid G1 phase nuclei, as supported by the presence of a significant population in the octoploid area (tetraploid G2 and octoploid G1 nuclei).

Unlike AAF, which blocks hepatocellular polyploidisation and thus expands the fraction of proliferating diploid hepatocytes (Seglen et al., 1988a; Saeter et al., 1988b), PB does not significantly change the hepatocellular ploidy distributions in normal (regenerated) liver (Seglen et al., 1988b). Some previous reports have even indicated that PB induces increased polyploidisation when stimulating the adult rat liver to grow (Stäubli et al., 1969; Argyris, 1974). The pronounced degree of diploidy seen in nearly all host liver tumours must therefore reflect a constitutive growth pattern which is not altered when tumour growth is stimulated by PB. PB would thus appear capable of stimulating polyploidising as well as non-polyploidising hepatocyte proliferation, the growth pattern being determined by the nature of the cells rather than by the promoter.

Hepatocellular polyploidisation is considered to be an irreversible process and a feature of cellular differentiation (Carriere, 1969; Brodsky & Uryvaeva, 1977). Assuming that this irreversibility holds true also for cells involved in the hepatocarcinogenic process, only diploid precursor cells can give rise to diploid tumours. In our model of primary hepatocarcinogenesis, the fraction of diploid hepatocytes is increased (by AAF) 4–5 times above normal at the preneoplastic stages (Schwarze et al., 1984; Seglen et al., 1988a; Saeter et al., 1988b). In DEN-initiated livers, the fraction of diploid hepatocytes remains significantly elevated even after AAF withdrawal, paralleling the retention of hepatocytes with elevated GGT levels (Seglen et al., 1988a). Some carcinogen-altered cells have thus come to express both phenotypic alterations in a constitutive manner. Subsequently developing neoplastic nodules and hepatocellular carcinomas are likewise predominantly diploid (Saeter et al., 1988a; Seglen et al., 1988a) as well as GGT-positive (our unpublished results), suggesting the possibility of a precursor–product relationship.

The present study shows that following transplantation of preneoplastic cells generated in this model to a new liver environment in syngeneic hosts, the resulting tumours are still predominantly diploid (and GGT-positive), regardless of the ploidy composition of the donor cell suspension. Accordingly, it is reasonable to assume that it is the diploid, GGT-positive cells present in all donor suspensions that give rise ot the host liver tumours. In the few tumours with small aneuploid subpopulations, the majority (75–85%) of nuclei were nevertheless diploid (Figure 6), indicating that the aneuploid clones have been formed by derangement of an already established diploid–diploid proliferation pattern.

These results illustrate the fundamental and constitutive nature of the switch in hepatocellular proliferation from a normal polyploidising programme to a non-polyploidising diploid divisional programme, seen at all stages in our model of experimental hepatocarcinogenesis. The reports of similar findings in other models (Neal et al., 1976; Irving et al., 1977; Styles et al., 1985; Deleener et al., 1987) may well indicate that this is a general feature of liver carcinogenesis.

This work was generously supported by The Norwegian Cancer Society.

### References

ARGYRIS, T.S. (1974). Stimulators, enzyme induction and the control of liver growth. In *Control of Proliferation in Animal Cells*, Clarkson, B. & Baserga, R. (eds) p. 49. Cold Spring Harbor Laboratory.

BLOBEL, G. & POTTER, V.R. (1966). Nuclei from rat liver: isolation method that combines purity with high yield. *Science*, **154**, 1662.

BRODSKY, W.Y. & URYVAEVA, I.V. (1977). Cell polyploidy: its relation to tissue growth and function. *Int. Rev. Cytol.*, **50**, 275.

CARRIERE, R. (1969). The growth of liver parenchymal nuclei and its endocrine regulation. *Int. Rev. Cytol.*, **25**, 201.

CONWAY, J.G., POPP, J.A. & THURMAN., R.G. (1985). Microcirculation of hepatic nodules from diethylnitrosamine-treated rats. *Cancer Res.*, **45**, 3620.

DELEENER, A., CASTELAIN, P., PREAT, V., GERLACHE, J.D., ALEXANDRE, H. & KIRSCH-VOLDERS, M. (1987). Changes in nodular transcriptional activity and nuclear DNA content during the first stages of rat hepatocarcinogenesis. *Carcinogenesis*, **8**, 195.

FINKELSTEIN, S.D., LEE, G., MEDLINE, A., TATEMATSU, M., MAKOWKA, L. & FARBER, E. (1983). An experimental method for rapid growth of liver in spleen. *Am. J. Pathol.*, **110**, 119.

HANIGAN, M.H. & PITOT, H.C. (1985). Growth of carcinogen-altered rat hepatocytes in the liver of syngeneic recipients promoted with phenobarbital. *Cancer Res.*, **45**, 6063.

HUNT, J.M., BUCKLEY, M.T., ONNINK, P.A., ROLFE, P.B. & LAISHES, B.A. (1982). Liver cell membrane alloantigens as cellular markers in genotypic mosaic rat livers undergoing chemically induced hepatocarcinogenesis. *Cancer Res.*, **42**, 227.

IRVING, C.C., ROSZELL, J.A. & FREDI, J.L. (1977). Effects of chronic feeding of 2-acetylaminofluorene on nuclear populations in rat liver. *Adv. Enzyme Regul.*, **16**, 365.

LAISHES, B.A., FINK, L. & CARR, B.I. (1980). A liver colony assay for a new hepatocyte phenotype as a step towards purifying new cellular phenotypes that arise during hepatocarcinogenesis. *Ann. NY Acad. Sci.*, **349**, 373.

LAISHES, B.A. & ROLFE, P.B. (1980). Quantitative assessment of liver colony formation and hepatocellular carcinoma incidence in rats receiving intravenous injections of isogeneic cells isolated during hepatocarcinogenesis. *Cancer Res.*, **40**, 4133.

NEAL, G.E., GODOY, H.M., JUDAH, D.J. & BUTLER, W.H. (1976). Some effects of acute and chronic dosing with aflatoxin B1 on rat liver nuclei. *Cancer Res.*, **36**, 1771.

ROOMI, M.W., HO, R.K., SARMA, D.S.R. & FARBER, E. (1985). A commonon biochemical pattern in preneoplastic hepatocyte nodules generated in four different models in the rat. *Cancer Res.*, **45**, 564.

RUTENBURG, A.M., KIM, H., FISHBEIN, J.W., HANKER, J.S. WASSERKRUG, H.L. & SELIGMAN, A.M. (1969). Histochemical and ultrastructural demonstration of γ-glutamyl transpeptidase activity. *J. Histochem. Cytochem.*, **17**, 517.

SAETER, G., SCHWARZE, P.E., NESLAND, J.M., JUUL, N., PETTERSEN, E.O. & SEGLEN, P.O. (1988a). The polyploidizing growth pattern of normal rat liver is replaced by divisional, diploid growth in hepatocellular nodules and carcinomas. *Carcinogenesis*, **9**, 939.

SAETER, G., SCHWARZE, P.E., NESLAND, J.M. & SEGLEN, P.O. (1987). Transplantation of preneoplastic rat hepatocytes by intraportal injection. *Toxicol. Pathol.*, **15,** 78.

SAETER, G., SCHWARZE, P.E. & SEGLEN, P.O. (1988*b*). Shift from polyploidizing to non-polyploidizing growth in carcinogen-treated rat liver. *J. Natl Cancer Inst.*, **80,** 950.

SCHULTE-HERMANN, R., TIMMERMANN-TROSIENER, I. & SCHUPPLER, J. (1982). Response of liver foci in rats to hepatic tumor promoters. *Toxicol. Pathol.*, **10,** 63.

SCHWARZE, P.E., PETTERSEN, E.O., SHOAIB, C. & SEGLEN, P.O. (1984). Emergence of a population of small, diploid hepatocytes during hepatocarcinogenesis. *Carcinogenesis*, **5,** 1267.

SCHWARZE, P.E., PETTERSEN, E.O., TOLLESHAUG, H. & SEGLEN, P.O. (1986). Isolation of carcinogen-induced diploid rat hepatocytes by centrifugal elutriation. *Cancer Res.*, **46,** 4732.

SCHWARZE, P.E. & SEGLEN, P.O. (1985). Reduced autophagic activity, improved protein balance and enhanced *in vitro* survival of hepatocytes isolated from carcinogen-treated rats. *Exp. Cell Res.*, **157,** 15.

SEGLEN, P.O. (1976). Preparation of isolated rat liver cells. *Meth. Cell Biol.*, **13,** 29.

SEGLEN, P.O. (1979). Disaggregation and separation of rat liver cells. In *Cell Populations* vol. 9, Reid, E. (ed) p. 25. Halsted Press: Chichester.

SEGLEN, P.O., SAETER, G. & SCHWARZE, P.E. (1988*a*). Nuclear alterations during hepatocarcinogenesis: Promotion by 2-acetylaminofluorene. In *Experimental Hepatocarcinogenesis*, Preat, V. & Roberfroid, M. (eds) p. 221. Plenum Press: London.

SEGLEN, P.O., SCHWARZE, P.E. & SAETER, G. (1986). Changes in cellular ploidy and autophagic responsiveness during rat liver carcinogenesis. *Toxicol. Pathol.*, **14,** 342.

SEGLEN, P.O., SCHWARZE, P.E. & SAETER, G. (1988*b*). Nuclear alterations in liver carcinogenesis: the role of non-polyploidizing growth. In *Chemical Carcinogenesis: Models and Mechanisms*, Feo, F., Pani, P. & Garcea, R. (eds). Plenum Press: New York.

SQUIRE, R.A. & LEVITT, M.H. (1975). Report of a workshop on classification of specific hepatocellular lesions in rats. *Cancer Res.*, **35,** 3214.

STÄUBLI, W., HESS, R. & WEIBEL, E.R. (1969). Correlated morphometric and biochemical studies on the liver cell. II. Effects on phenobarbital on rat hepatocytes. *J. Cell. Biol.*, **2,** 92.

STYLES, J., ELLIOT, B.M., LEFEVRE, P.A. & 4 others (1985). Irreversible depression in the ratio of tetraploid:diploid liver nuclei in rats treated with 3′-methyl-4-dimethylaminoazobenzene (3′M). *Carcinogenesis*, **6,** 21.

VINDELØV, L.L., CHRISTENSEN, I.J. & NISSEN, N.I. (1983). A detergent-trypsin method for the preparation of nuclei for flow cytometric DNA analysis. *Cytometry*, **3,** 323.

WATANABE, K. & WILLIAMS, G.M. (1978). Enhancement of rat hepatocellular-altered foci by the liver tumor promoter phenobarbital: evidence that foci are precursors of neoplasms and that the promoter acts on carcinogen-induced lesions. *J. Natl Cancer Inst.*, **61,** 1311.

WEISS, L., WARD, P.M. & HOLMES, J.C. (1983). Liver-to-lung traffic of cancer cells. *Int. J. Cancer*, **32,** 79.

ZAWYDIWSKI, R. & DUNCAN, G.R. (1978). Spontaneous $^{51}$Cr release by isolated rat hepatocytes: an indicator of membrane damage. *In Vitro*, **14,** 707.